# Effect of prophylactic noninvasive oxygen therapy after planned extubation on extubation failure in high-risk patients: a retrospective propensity score-matched cohort study

**DOI:** 10.3389/fmed.2024.1481083

**Published:** 2024-09-26

**Authors:** Xiaozhuo Zheng, Lixiong Lu, Mengyi Ma, Xiaofeng Lei

**Affiliations:** ^1^Department of Anesthesiology, Women and Children’s Hospital of Chongqing Medical University, Chongqing, China; ^2^Department of Anesthesiology, Chongqing Health Center for Women and Children, Chongqing, China; ^3^Department of Pulmonary and Critical Care Medicine, The First Affiliated Hospital of Chongqing Medical University, Chongqing, China

**Keywords:** noninvasive oxygen therapy, extubation failure, high-risk patients, delirium, noninvasive ventilation, high-flow nasal cannula

## Abstract

**Background:**

Extubation failure (EF) is common in the intensive care unit (ICU) and is associated with poor prognosis, especially in high-risk patients. However, the efficacy of prophylactic noninvasive oxygen therapy (NIT), including noninvasive ventilation (NIV) and high-flow nasal cannula (HFNC), in reducing EF in high-risk patients remains controversial. Therefore, we aimed to evaluate the effect of post-extubation prophylactic NIT on EF in high-risk patients.

**Methods:**

This was a retrospective observational study conducted in the ICU from March 2018 to December 2023. We included adult patients at high risk for reintubation who were mechanically ventilated for over 24 h and successfully passed the spontaneous breathing trial (SBT). Immediately after extubation, patients underwent NIT or conventional oxygenation therapy (COT). The primary outcome was the EF rate within 7 days after extubation.

**Results:**

There were 440 patients in the NIT group and 274 in the COT group. After propensity-score matching, 227 subjects were enrolled in each group. NIT reduced the rate of EF (18.0% vs. 34.3%, *p* < 0.001) and reintubation (10.5% vs. 18.2% *p* = 0.003) compared with COT, which was confirmed in propensity-matched cohort (17.6% vs. 32.2%, *p* < 0.001; 11.5% vs. 19.8%, *p* = 0.014). Multivariate logistic regression analysis indicated that prophylactic NIT (*p* = 0.001) and higher ROX index (*p* = 0.022) were associated with reduced risk of EF. While higher fluid balance (*p* = 0.013), higher RSBI (*p* < 0.001), and the occurrence of delirium (*p* = 0.032) may be the risk factors for EF. Subgroup analysis showed that post-extubation NIT was more effective in elderly patients, and HFNC was non-inferior to NIV in reducing EF. While HFNC had a tendency to reduce the incidence of delirium.

**Conclusion:**

Post-extubation prophylactic NIT is effective in reducing EF in high-risk patients, especially in the elderly patients. HFNC is an alternative treatment to NIV. Fluid balance, RSBI, ROX index, and delirium are associated with the occurrence of EF.

## 1 Introduction

Extubation failure (EF) is common in the intensive care unit (ICU) and still occurs in 10–20% of mechanically ventilated patients who successfully complete the spontaneous breathing trial (SBT) (1). Among high-risk patients, that is, those older than 65 years or with any underlying chronic cardiac or respiratory disease, the EF rate is even as high as 48% (2). EF increases mortality by 25–50% and prolongs ICU stay and length of hospital stay (LOS) (3). Therefore, it is necessary to provide effective post-extubation respiratory support to prevent the occurrence of EF.

In addition to high-risk factors, delirium may also cause EF. Delirium is frequent in the ICU and may contribute to EF through altered consciousness, agitation and subsequent sedation, aspiration, and intolerance to noninvasive mechanical ventilation (NIV) (4). A reintubation rate of 22% has been reported among patients who developed delirium on the day of extubation (5). Identification of risk factors for EF is particularly important in predicting the occurrence of EF and reintubation.

NIV has been recommend for patients with hypercapnia. However, the effect of prophylactic use on reintubation and mortality in high-risk patients remains controversial (6, 7). In addition, NIV is susceptible to gastric distention, skin damage and claustrophobia, limiting its widespread use and reducing its efficacy in EF (8, 9). In contrast, high-flow nasal cannula (HFNC) improves patient comfort and tolerability (10). HFNC has also been reported to suppress delirium, which is a contributing factor to reintubation (11). In clinical practice, HFNC has emerged as a promising treatment strategy for patients with hypoxemic respiratory failure. In high-risk patients, HFNC was even comparable to NIV in preventing EF and reintubation (12). In recent years, an increasing number of studies referred to NIV and HFNC collectively as noninvasive oxygen therapy (NIT), and investigated its efficacy in ICU patients (13, 14).

A relevant randomized controlled trial (RCT) indicated that preventive use of NIT did not prevent reintubation compared with conventional oxygen therapy (COT) (15). However, the population was unselected and the efficacy of NIT in high-risk patients is unclear. Therefore, we conducted this retrospective observational cohort study to evaluate the efficacy of post-extubation prophylactic NIT to reduce EF in high-risk patients and to identify potential risk factors for EF.

## 2 Materials and methods

This was a retrospective observational cohort study conducted in the ICU of the First Affiliated Hospital of Chongqing Medical University from March 2018 to December 2023. The study was approved by the institutional ethics committee of the First Affiliated Hospital of Chongqing Medical University and registered with the Chinese Clinical Trial Registry (ChiCTR2200061820). Informed consent was waived because of the retrospective observational nature of the study. All records and data were anonymized and de-identified prior to analysis.

### 2.1 Study population

We reviewed the records of all adult patients (≥ 18 years) admitted to the ICU and receiving MV for at least 24 h. In further screening, patients at high-risk of reintubation (7) who successfully passed the SBT and received post-extubation prophylactic NIV or HFNC and COT were included in the study. Patients were considered with high risk factors for reintubation if they fulfilled any of the following criteria as described in earlier studies: (1) age over 65 years; (2) had any underlying chronic cardiac or pulmonary disease. Underlying chronic cardiac diseases included left ventricular dysfunction, defined as left ventricular ejection fraction ≤ 45%; history of cardiogenic pulmonary edema; documented ischemic heart disease; or permanent atrial fibrillation. Underlying chronic pulmonary diseases included chronic obstructive pulmonary disease (COPD), obesity-hypoventilation syndrome, or restrictive pulmonary disease.

Exclusion criteria were as follows: (1) died before SBT or accidental extubation; (2) tracheotomy before weaning attempt; (3) intervention lasted less than 24 hours; (4) post-extubation surgery; (5) refusal of resuscitation and reintubation; (6) missing data.

### 2.2 Interventions

Patients who received prophylactic NIV (BiPAP Vision, Philips Respironics, USA) immediately after extubation were classified as the NIT group. The course of NIV was at least 24 h, but could be interrupted by drinking, feeding, and clearing secretions. Depending on patient respiratory status, NIV could be continued until complete recovery. Positive end-expiratory pressure (PEEP) was set at 4–6 cmH_2_O, and pressure-support level was initially set at 8 cmH_2_O (titrated 1–2 cmH_2_O) to obtain a tidal volume of about 6–8 mL/kg. Fractional inspiratory oxygen ratio (FiO_2_) was adjusted to maintain peripheral capillary oxygen saturation (SpO_2_) above 92%.

Patients in the NIT group could also be treated with HFNC (Optiflow, Fisher and Paykel Healthcare, Canada) immediately after extubation for at least 24 h. Flow was initially set at 10 L/min and titrated upwards in 5 L/min steps until patients experienced discomfort. FiO_2_ was adjusted to maintain SpO_2_ above 92%. To provide sufficient humidification, the temperature of the heated humidifier was set to 37°C.

Patients in the control group received COT via face mask or nasal cannula. FiO_2_ was set to achieve SpO_2_ over 92%. And COT was administered according to patient needs.

### 2.3 Study outcomes

The primary outcome was the rate of EF within 7 days following extubation. EF was defined as the need for reintubation or NIV rescue treatment (16). Secondary outcomes included reintubation within 7 days after extubation (2, 12), post-extubation respiratory failure (7), delirium on the day of extubation (17), in-hospital mortality, and post-extubation ICU stay and LOS. Patients were immediately reintubated if any of the following criteria was met: massive aspiration, uncontrollable agitation, sputum retention, hemodynamic deterioration unresponsive to vasoactive drugs, respiratory pauses with loss of consciousness or gasping for air, heart rate < 50 beats per min with loss of alertness, and cardiac or respiratory arrest. And respiratory failure was defined as the presence of any of the criteria below: respiratory rate > 35 breaths per minute, clinical signs suggesting respiratory distress, respiratory acidosis (pH < 7.35 and PaCO2 > 45 mmHg), hypoxemia (SpO2 ≤ 90% or PaO2:FiO2 ratio ≤ 120 mmHg at FIO2 > 0.4), decreased level of consciousness (GCS > 1 point decrease), and agitation. Delirium was defined as a disturbance of consciousness characterized by a sudden onset and a fluctuating course of attention accompanied by a change in perception or cognition. Delirium was routinely measured by ICU nurses using the Confusion Assessment Method for the ICU (CAM-ICU).

### 2.4 Data collection

The following data were collected retrospectively from the medical records: age, gender, underlying diseases, main reason for intubation, acute physiology and chronic health evaluation II (APACHE II) score at ICU admission and at extubation, duration of MV before extubation, fluid balance and secretion volume 24h before extubation, use of vasopressors at extubation, hemoglobin, and Glasgow Coma Score (GCS). Vital signs and arterial blood gas parameters were collected before SBT and at extubation, including mean arterial pressure, heart rate, respiratory rate, tidal volume, SpO_2_, rapid shallow breathing index (RSBI), the ratio of SpO_2_/FiO_2_ to respiratory rate (ROX index), as well as pH, partial pressure of oxygen (PaO_2_), partial pressure of carbon dioxide (PaCO_2_), the ratio of PaO_2_ to FIO_2_ (oxygenation index).

### 2.5 Subgroup analysis

Patients were divided into two subgroups based on age (> 65 years and ≤ 65 years) to demonstrate the impact of prophylactic NIT on EF, particularly in elderly high-risk patients. And another subgroup analysis was performed in the NIT group to determine whether HFNC was noninferior to NIV in reducing EF rate.

### 2.6 Statistical analysis

Due to the retrospective design of the study, propensity score matching (PSM) was performed to reduce the effects of selection bias and possible confounding factors between the two groups. The following variables were selected to generate the propensity score: age, gender, underling diseases, intubation period, APACHE II score at ICU admission and at extubation, fluid volume, secretion volume, hemoglobin, GCS, and physiological parameters before SBT and at extubation. After calculating the propensity scores, we matched patients with similar propensity scores in each group in a 1:1 ratio using the nearest neighbor method, with the caliper width set to 0.1.

Data were summarized as mean ± standard deviation (SD) or median (25th percentile, 75th percentile) depending on distribution. The Mann-Whitney U test was used for group comparisons of continuous variables when the data were abnormally distributed; otherwise, Student’s t-test was applied. Categorical variables were expressed as numbers (percentage) and compared using the chi-square test or Fisher’s exact test as appropriate. Univariate logistic regression analysis was used to identify independent factors related to EF within 7 days after extubation. Variables with *p* < 0.1 in the univariate analysis and other clinically significant variables were included in the conditional stepwise multivariable logistic regression. All statistical tests were 2-sided and p-values < 0.05 were considered statistically significant. Statistical analysis was performed using IBM SPSS Statistics 26.0 (SPSS Inc., Chicago, IL, USA).

## 3 Results

### 3.1 Patient characteristics

**FIGURE 1:**
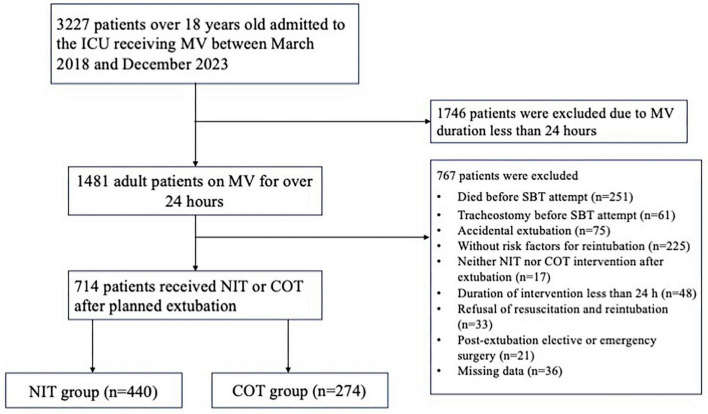
The flow diagram of study population. ICU, intensive care unit; MV, mechanical ventilation; SBT, spontaneous breathing trial; COT, conventional oxygen therapy; NIT, noninvasive oxygen therapy.

Between March 2018 and December 2023, 3227 patients over 18 years old were admitted to the ICU receiving MV. Of these, 1746 patients were excluded due to MV duration less than 24 h. Among the remaining 1481 patients, 767 were excluded for the following reasons: death (*n* = 251) or tracheostomy (*n* = 61) before SBT attempt, accidental extubation (*n* = 75), without risk factors for reintubation (*n* = 225), receiving neither NIT nor COT intervention immediately after extubation (*n* = 17), duration of intervention less than 24 h (*n* = 48), refusal of resuscitation and reintubation (*n* = 33), post-extubation surgery (*n* = 21) and loss of information (*n* = 36). Overall, we analyzed data from 714 patients, including 440 patients in the NIT group and 274 patients in the COT group. The flow diagram is shown in Figure 1.

The baseline characteristics of both groups are presented in Table 1. There were more male patients in the NIT group than in the COT group (75.5% vs. 67.2%, p = 0.016). The NIT group had a higher proportion of patients with acute exacerbation of chronic obstructive pulmonary disease (AECOPD) (52.0% vs. 21.9%, *p* < 0.001), and a lower proportion of patients with pneumonia or acute respiratory distress syndrome (ARDS) than the COT group (42.5% vs. 57.3%, *p* < 0.001). Patients receiving NIT had a longer intubation period than those in the COT group [6 (4, 10) vs. 5.5 (3, 7) d, *p* = 0.001]. The amount of secretion 24 h before extubation was significantly higher in the NIT group than in the COT group [64 (38, 78) vs. 41 (21, 61) ml, *p* < 0.001]. The pre-SBT tidal volume was greater in the NIT group compared to the control group (474.0 ± 83.1 vs. 450.9 ± 78.3 ml, *p* < 0.001). Patients receiving prophylactic NIT had higher PaCO_2_ levels before SBT (48.7 ± 11.6 vs. 41.4 ± 9.4 mmHg, *p* < 0.001) and at extubation (47.1 ± 9.5 vs. 40.1 ± 5.9 mmHg, *p* < 0.001) than those in the COT group. After a 1:1 PSM, 227 matched subjects were included in each group. There were no significant differences in demographic and clinical characteristics between the two matched cohorts, except that patients in the NIT group had a higher secretion volume 24h before extubation (shown in Table 1).

**TABLE 1:** Baseline characteristics of study population.

	Overall cohort			Propensity-matched cohort		
	COT *n* = 274	NIT *n* = 440	*P*-value	COT *n* = 227	NIT *n* = 227	*P*-value
Age (years)	69.3 ± 14.9	71.0 ± 11.6	0.101	69.9 ± 14.5	71.4 ± 11.2	0.219
Male	184 (67.2)	332 (75.5)	0.016	159 (70.0)	170 (74.9)	0.248
**Underlying diseases**						
*AECOPD*	60 (21.9)	229 (52.0)	< 0.001	49 (21.6)	64 (28.2)	0.103
*Pneumonia/ARDS*	157 (57.3)	187 (42.5)	< 0.001	144 (63.4)	137 (60.4)	0.499
*CVD*	124 (45.3)	219 (49.8)	0.240	117 (51.5)	128 (56.4)	0.300
*Hypertension*	140 (51.1)	198 (45.0)	0.113	86 (37.9)	95 (41.9)	0.388
*Diabetes*	82 (29.9)	108 (24.5)	0.114	63 (27.8)	65 (28.6)	0.835
*Liver disease*	34 (12.4)	42 (9.5)	0.228	16 (7.0)	12 (5.3)	0.435
*Renal disease*	40 (14.6)	61 (13.9)	0.784	26 (11.5)	36 (15.9)	0.172
*Shock*	81 (29.6)	130 (29.5)	0.996	57 (25.1)	48 (21.1)	0.316
*Arrhythmia*	11 (4.0)	19 (4.3)	0.844	8 (3.5)	15 (6.6)	0.134
*PE*	10 (3.6)	25 (5.7)	0.221	7 (3.1)	2 (0.9)	0.175
*Asthma*	9 (3.3)	13 (3.0)	0.804	4 (1.8)	7 (3.1)	0.360
APACHE II score at ICU admission	21.4 ± 5.6	21.3 ± 5.8	0.880	21.3 ± 5.5	21.2 ± 6.1	0.796
APACHE II score at extubation	14.1 ± 3.2	14.3 ± 3.2	0.390	14.1 ± 3.1	14.6 ± 3.1	0.083
Intubation period (days)	5.5 (3,7)	6 (4,10)	0.001	6 (3,7.5)	5 (4,9)	0.063
Main reason for intubation			0.063			0.477
*Acute respiratory failure*	145 (52.9)	260 (59.1)		126 (55.5)	139 (61.2)	
*Coma*	55 (20.1)	76 (13.6)		44 (19.4)	40 (17.6)	
*Acute heart failure*	3 (1.1)	12 (2.7)		2 (0.9)	6 (2.6)	
*Surgery*	29 (10.6)	32 (7.3)		21 (9.3)	17 (7.5)	
*Airway protection*	35 (12.8)	55 (12.5)		28 (12.3)	21 (9.3)	
*Other*	8 (2.9)	5 (1.1)		6 (2.6)	4 (1.8)	
Fluid balance 24h before extubation (ml)	688 (−325,1219)	345 (−297,1070)	0.010	619 (−325,1219)	381 (−318,1029)	0.247
Secretion volume 24h before extubation (ml) ^†^	41 (21,61)	64 (38,78)	< 0.001	42 (21,61)	62 (47,75)	< 0.001
Use of vasopressors at extubation	40 (14.6)	52 (11.8)	0.281	32 (14.1)	19 (8.4)	0. 053
Hemoglobin (g/dL)	103.4 ± 4.7	102.2 ± 3.9	0.182	104.1 ± 4.0	103.4 ± 3.9	0.216
GCS	14.1 ± 1.3	14.5 ± 1.6	0.327	14.2 ± 1.7	14.1 ± 1.2	0.673
**Physiological parameters before SBT**						
*Mean blood pressure (mmHg)*	88.8 ± 13.3	89.8 ± 12.9	0.303	88.9 ± 12.8	88.7 ± 13.4	0.915
*Heart rate (beats/min)*	90.4 ± 14.1	90.7 ± 15.7	0.813	91.0 ± 14.0	91.0 ± 14.2	0.979
*Respiratory rate (breaths/min)*	20.4 ± 4.3	20.4 ± 6.0	0.868	20.5 ± 4.2	20.9 ± 4.3	0.365
*Tidal volume (ml)*	450.9 ± 78.3	474.0 ± 83.1	< 0.001	459.0 ± 78.6	464.6 ± 81.8	0.457
*SpO_2_ (%)*	98.2 ± 1.3	97.9 ± 1.7	0.020	98.1 ± 1.3	97.9 ± 1.9	0.419
*pH*	7.4 ± 0.1	7.4 ± 0.1	0.022	7.4 ± 0.1	7.4 ± 0.0	0.484
*PaO_2_ (mmHg)*	120.5 ± 33.9	113.8 ± 26.7	0.005	118.4 ± 33.9	114.0 ± 22.4	0.107
*PaCO_2_ (mmHg)*	41.4 ± 9.4	48.7 ± 11.6	< 0.001	42.5 ± 9.1	43.5 ± 10.2	0.252
*PaO_2_/FiO_2_*	274.0 ± 77.6	262.2 ± 66.5	0.037	271.0 ± 75.7	262.4 ± 71.5	0.214
*RSBI*	46.6 ± 13.3	44.3 ± 14.2	0.033	46.1 ± 13.1	46.3 ± 13.2	0.874
*ROX index*	11.8 ± 4.0	11.9 ± 3.8	0.921	11.8 ± 4.0	11.3 ± 3.4	0.127
SBT protocol			0.132			0.125
*PSV*	228 (83.2)	384 (87.3)		192 (84.6)	203 (89.4)	
*PS*	10.5 ± 3.4	12.0 ± 4.0	< 0.001	10.6 ± 3.6	11.0 ± 3.7	0.425
*PEEP*	5.4 ± 1.6	5.8 ± 1.7	0.002	5.4 ± 1.7	5.6 ± 1.8	0.279
*T-Piece*	46 (16.8)	56 (12.7)		35 (15.4)	24 (10.6)	
**Physiological parameters at extubation**						
*Mean blood pressure (mmHg)*	87.7 ± 10.5	87.8 ± 12.3	0.879	87.1 ± 10.2	88.2 ± 11.7	0.302
*Heart rate (beats/min)*	89.9 ± 10.4	91.4 ± 12.7	0.083	90.2 ± 10.6	91.9 ± 13.0	0.141
*Respiratory rate (breaths/min)*	20.2 ± 3.6	20.6 ± 3.8	0.271	20.1 ± 3.6	20.6 ± 3.7	0.148
*Tidal volume (ml) ^‡^*	406.6 ± 84.0	412 ± 63.8	0.404	409.9 ± 89.3	412.7 ± 61.7	0.716
*SpO_2_, %*	97.3 ± 1.8	97.0 ± 1.9	0.013	97.3 ± 1.9	97.1 ± 2.0	0.225
*pH*	7.4 ± 0.0	7.4 ± 0.0	0.112	7.4 ± 0.0	7.4 ± 0.0	0.306
*PaO_2_ (mmHg)*	106.9 ± 36.4	102.1 ± 28.5	0.065	105.6 ± 33.4	103.3 ± 29.5	0.444
*PaCO_2_ (mmHg)*	40.1 ± 5.9	47.1 ± 9.5	< 0.001	41.0 ± 5.9	42.0 ± 6.5	0.068
*PaO_2_/FiO_2_*	258.4 ± 83.7	253.9 ± 76.0	0.466	256.7 ± 80.8	258.3 ± 77.4	0.830
*RSBI ^§^*	51.4 ± 14.9	50.6 ± 12.9	0.542	50.9 ± 15.3	50.1 ± 11.6	0.562
*ROX index*	12.2 ± 2.8	12.2 ± 3.1	0.838	12.3 ± 2.9	12.3 ± 2.9	0.836

COT, conventional oxygen therapy; NIT, non-invasive oxygen therapy; AECOPD, acute exacerbation of chronic obstructive pulmonary disease; ARDS, acute respiratory distress syndrome; CVD, cardiovascular disease; PE, pulmonary embolism; APACHE II, acute physiology and chronic health evaluation II; SBT, spontaneous breathing trial; GCS, Glasgow Coma Score; RSBI, rapid shallow breathing index; ROX, the ratio of SpO_2_/FiO_2_ to respiratory rate; PSV, pressure support ventilation; PS, pressure support; PEEP, positive end-expiratory pressure.

^†^Secretion volume was available for 368 patients in the overall cohort: 128 in the COT group and 240 in the NIT group. ^‡^Pre-extubation tidal volume was available for 612 patients in the overall cohort: 228 in the COT group and 384 in the NIT group. Pre-extubation tidal volume was available for 198 patients in the propensity-matched cohort: 102 in the COT group and 96 in the NIT group. ^§^Pre-extubation RSBI was available for 612 patients in the overall cohort: 228 in the COT group and 384 in the NIT group. Pre-extubation RSBI was available for 198 patients in the propensity-matched cohort: 102 in the COT group and 96 in the NIT group.

### 3.2 Primary outcome

The occurrence of EF in both groups is summarized in Table 2. Of the 440 patients treated with prophylactic NIT, 79 failed to extubate, with a lower incidence than the control group (18.0% vs. 34.3%, *p* < 0.001). In the propensity-matched cohort, the EF rate in the COT group was 32.2%, nearly 2 times that of the NIT group (*p* < 0.001).

**TABLE 2:** Patient outcomes in the NIT and COT group.

	Overall cohort			Propensity-matched cohort		
	COT *n* = 274	NIT *n* = 440	*P*-value	COT *n* = 227	NIT *n* = 227	*P*-value
Extubation failure	94 (34.3)	79 (18.0)	< 0.001	73 (32.2)	40 (17.6)	< 0.001
Reintubation	50 (18.2)	46 (10.5)	0.003	45 (19.8)	26 (11.5)	0.014
Respiratory failure	107 (39.1)	192 (43.6)	0.227	81 (35.7)	99 (43.6)	0.084
Delirium	142 (51.8)	249 (56.6)	0.213	118 (52.0)	137 (60.4)	0.089
In-hospital mortality	76 (27.9)	77 (17.5)	0.001	60 (26.4)	45 (19.8)	0.095
Post-extubation ICU stay (days)	5 (3,9)	7 (4,11)	< 0.001	5 (3,12)	5 (4,11)	0.068
Post-extubation LOS (days)	7 (4,16)	9 (6,15)	0.004	9 (5,17)	10 (5,16.5)	0.114

COT, conventional oxygen therapy; NIT, noninvasive oxygen therapy; ICU, intensive care unit; LOS, length of hospital stay.

### 3.3 Secondary outcomes

In the overall cohort, NIT was associated with a lower reintubation rate (10.5% vs. 18.2%, *p* = 0.003) and in-hospital mortality (17.5% vs. 27.9%, *p* = 0.001) compared with COT. However, the incidence of respiratory failure and delirium were comparable between the two groups (39.1% vs. 43.6%, *p* = 0.227; 51.8% vs. 56.6%, *p* = 0.213). As shown in Table 2, NIT group had longer post-extubation ICU stay and post-extubation LOS than the control group [7(4, 11) vs. 5(3, 9) d, *p* < 0.001; 9 (6, 15) vs. 7 (4, 16) d, *p* = 0.004, respectively]. In the propensity-matched cohort, NIT reduced the incidence of reintubation compared with the COT (11.5% vs. 19.8%, *p* = 0.014). However, there were no significant differences in respiratory failure, delirium, in-hospital mortality, post-extubation ICU stay, and post-extubation LOS between the two matched cohorts.

### 3.4 Subgroup analysis

Among the 714 patients in the study, 512 were over 65 years old (as shown in Table 3). In this subgroup, NIT reduced the rate of EF (16.3% vs. 38.0%, *p* < 0.001) and reintubation (12.5% vs. 23.0%, *p* = 0.002) compared with COT, as confirmed in the propensity-matched cohort (13.9% vs. 37.3%, *p* < 0.001; 8.9% vs. 24.7%, *p* < 0.001, respectively). In both cohorts, there were no differences in respiratory failure, delirium, post-extubation ICU stay or post-extubation LOS between the two groups. In the non-elderly high-risk subgroup (*n* = 202), NIT was not superior to COT in reducing EF, reintubation, respiratory failure, delirium, and in-hospital mortality, as demonstrated in the propensity-matched cohort. The LOS after extubation was 10.5d in the NIT group, 3.5d longer than in the COT group (*p* = 0.002). And in the propensity-matched cohort, NIT also prolonged post-extubation LOS compared with the control group [13 (9, 14) vs. 9 (3, 10)d, *p* = 0.017).

**TABLE 3:** Outcomes of patients > 65 years and ≤ 65 years of each group.

	Overall cohort			Propensity-matched cohort		
	COT *n* = 200	NIT *n* = 312	*P*-value	COT *n* = 166	NIT *n* = 158	*P*-value
**Patients > 65 years (*n* = 512)**						
Extubation failure	76 (38.0)	51 (16.3)	< 0.001	62 (37.3)	22 (13.9)	< 0.001
Reintubation	46 (23.0)	39 (12.5)	0.002	41 (24.7)	14 (8.9)	< 0.001
Respiratory failure	83 (41.5)	140 (44.9)	0.453	70 (42.2)	78 (49.4)	0.194
Delirium	127 (63.5)	218 (69.9)	0.133	105 (63.3)	113 (65.2)	0.113
In-hospital mortality	65 (32.5)	67 (21.5)	0.005	51 (35.5)	35 (18.4)	0.081
Post-extubation ICU stay (days)	6 (3,14)	7 (4,11)	0.092	5 (3,14.8)	8 (5,15)	0.179
Post-extubation LOS (days)	7 (4, 17)	9 (5, 14)	0.402	8.5 (4.3, 17.8)	10 (7, 17.5)	0.422
	**Overall cohort**			**Propensity-matched cohort**		
	**COT** ***n* = 74**	**NIT** ***n* = 128**	***P*-value**	**COT** ***n* = 61**	**NIT** ***n* = 69**	***P*-value**
**Patients ≤ 65 years (*n* = 202)**						
Extubation failure	18 (24.3)	28 (21.9)	0.689	11 (18.0)	18 (26.1)	0.271
Reintubation	4 (5.4)	7 (5.5)	0.985	4 (6.6)	12 (17.4)	0.061
Respiratory failure	24 (32.4)	52 (40.6)	0.247	11 (18.0)	21 (30.4)	0.101
Delirium	15 (20.3)	31 (24.2)	0.519	13 (21.3)	24 (34.8)	0.089
In-hospital mortality	11 (14.9)	10 (7.8)	0.114	9 (14.8)	10 (14.5)	0.966
Post-extubation ICU stay (days)	4 (2, 6.5)	6.5 (4, 9)	0.003	4 (2, 6)	6 (3, 13)	0.156
Post-extubation LOS (days)	7 (3, 9.5)	10.5 (6, 17)	0.002	9 (3, 10)	13 (9, 14)	0.017

COT, conventional oxygen therapy; NIT, noninvasive oxygen therapy; ICU, intensive care unit; LOS, length of hospital stay.

In the NIT group, 392 patients received prophylactic NIV after planned extubation and 48 patients received prophylactic HFNC. As illustrated in Table 4, HFNC was noninferior to NIV in reducing EF (*p* = 0.162), reintubation (*p* = 0.624), respiratory failure (*p* = 0.771), in-hospital mortality (*p* = 0.083), and shortening post-extubation ICU stay (*p* = 0.393) and post-extubation LOS (*p* = 0.754), which was also confirmed in the propensity-matched cohort. However, patients receiving HFNC had a lower incidence of delirium than those with NIV (35.4% vs. 59.2%, *p* = 0.002). After PSM, the rate of delirium was comparable between the two groups (46.7% vs. 62.4%, *p* = 0.100).

**TABLE 4:** Outcomes of patients receiving NIV and HFNC in the NIT group.

	Overall cohort			Propensity-matched cohort		
	NIV *n* = 392	HFNC *n* = 48	*P*-value	NIV *n* = 197	HFNC *n* = 30	*P*-value
Extubation failure	66 (16.8)	12 (25.0)	0.162	36 (18.3)	4 (13.3)	0.508
Reintubation	40 (10.2)	6 (12.5)	0.624	24 (12.2)	2 (6.7)	0.543
Respiratory failure	172 (43.9)	20 (41.7)	0.771	81 (41.1)	8 (26.7)	0.131
Delirium	232 (59.2)	17 (35.4)	0.002	123 (62.4)	14 (46.7)	0.100
In-hospital mortality	72 (18.4)	4 (8.3)	0.083	29 (14.7)	1 (3.3)	0.143
Post-extubation ICU stay (days)	7 (4, 11)	6.5 (5, 13.8)	0.393	8 (4, 13)	8 (5, 17)	0.628
Post-extubation LOS (days)	9 (6, 14)	9 (5, 18.5)	0.754	11 (7, 17)	17 (8, 19)	0.358

NIT, noninvasive oxygen therapy; ICU, intensive care unit; LOS, length of hospital stay; NIV, noninvasive mechanical ventilation; HFNC, high-flow nasal cannula.

### 3.5 Risk factors for EF

Univariate logistic regression analysis showed that there were significant differences in APACHE II score at extubation, fluid balance volume, secretion volume, intervention protocol, pre-SBT PaCO_2_, ROX index, RSBI, PEEP and delirium between the failed extubation group and the successful extubation group. After the above variables were inserted into the multivariable logistic regression analysis (as shown in Table 5), we found that prophylactic NIT was a protective factor for EF, both in the overall cohort (odds ratio [OR] = 0.20, 95% confidence interval [CI]: 0.06–0.73, *p* = 0.014) and in the propensity-matched cohort (OR = 0.06, 95% CI: 0.01–0.30, *p* = 0.001). Higher fluid balance 24h before extubation (OR = 1.02, 95% CI: 1.00–1.03, *p* = 0.002, for the overall cohort; OR = 1.01, 95% CI: 1.00–1.02, *p* = 0.013, for the propensity-matched cohort) and higher pre-extubation RSBI (OR = 1.19, 95% CI: 1.10–1.28, *p* < 0.001, for the overall cohort; OR = 1.17, 95%CI: 1.10–1.24, *p* < 0.001, for the propensity-matched cohort) were associated with an increased risk of EF. Delirium on the day of extubation appeared to be a risk factor for EF, both in the overall cohort (OR = 1.96, 95% CI: 1.27–2.54, *p* = 0.029) and in the propensity-matched cohort (OR = 1.78, 95% CI: 1.32–1.94, *p* = 0.032). Higher pre-SBT ROX index appeared to be a protective factor against EF (OR = 0.80, 95% CI: 0.64–1.00, *p* = 0.045, for the overall cohort; OR = 0.59, 95% CI: 0.37–0.93, *p* = 0.022, for the propensity-matched cohort).

**TABLE 5:** Multivariate logistic regression analyses identify risk factors for extubation failure.

	Overall cohort		Propensity-matched cohort	
	**OR (95%CI)**	***P*-value**	**OR (95%CI)**	***P*-value**
Prophylactic NIT	0.20 (0.06, 0.73)	0.014	0.06 (0.01, 0.30)	0.001
Fluid balance (ml)	1.02 (1.00, 1.03)	0.002	1.01 (1.00, 1.02)	0.013
Secretion volume (ml)	0.98 (0.96, 1.00)	0.010	0.97 (0.95, 0.99)	0.005
Pre-SBT ROX index	0.80 (0.64, 1.00)	0.045	0.59 (0.37, 0.93)	0.022
Pre-SBT RSBI	0.95 (0.90, 1.00)	0.033	0.95 (0.89, 1.01)	0.076
Pre-extubation RSBI	1.19 (1.10, 1.28)	<0.001	1.17 (1.10, 1.24)	<0.001
Delirium	1.96 (1.27, 2.54)	0.029	1.78 (1.32, 1.94)	0.032

NIT, noninvasive oxygen therapy; SBT, spontaneous breathing trial; RSBI, rapid shallow breathing index; ROX, the ratio of SpO_2_/FiO_2_ to respiratory rate.

## 4 Discussion

In this cohort study, prophylactic NIT (including NIV and HFNC) was superior to COT in reducing the rate of EF within 7 days after extubation in patients at high-risk of reintubation, especially in those older than 65 years. HFNC was noninferior to NIV in high-risk patients. In addition, higher fluid balance 24 h before extubation, lower pre-SBT ROX index, higher pre-extubation RSBI, and delirium on the day of extubation increased the risk of EF.

NIT was associated with a lower incidence of EF and reintubation in high-risk patients. The high success rate may be due to the superiority of NIV and HFNC over COT. As we know, NIV administered following extubation opens the upper airway, prevents alveolar collapse, and improves oxygenation (18). In high-risk patients, inspiratory positive airway pressure (IPAP) can reduce respiratory workload and compensate for increased airway resistance (19). Expiratory positive airway pressure (EPAP) increases end-expiratory lung volume and decreases venous return, especially in patients with congestive heart failure (20). Compared with COT, HFNC provides more predictable FiO_2_ and preserves the mucosal function (21). In addition, HFNC generates a positive airway pressure (between 2 and 8 cmH_2_O at the pharyngeal level) similar to positive end-expiratory pressure (PEEP), which may benefit high-risk patients (22–24).

However, a recent RCT indicated that the application of NIT after extubation was not able to prevent reintubation compared with usual care, contrary to our results (15). The different findings may be related to the study population. In the study by Casey et al. (15), critically ill adult patients undergoing MV were included, whereas we only focused on mechanically ventilated patients with high-risk factors for reintubation. Moreover, in that study (15), patients in the usual-care group could also be treated with NIV or HFNC at the discretion of the attending physicians, which may reduce the occurrence of reintubation. Furthermore, in the study by Casey et al. (15), HFNC was predominantly used in the NIT group, which may influence the efficacy of NIT. Therefore, more studies are needed to investigate the effectiveness of NIT on reintubation and EF.

In the subgroup analysis, NIT immediately after extubation benefited elderly patients, which was consistent with a cohort study (25). However, we found no effect of NIT on EF and reintubation in non-elderly high-risk patients compared with COT. In fact, age is an important factor in reintubation (26). In addition to being older than 65 years, elderly patients are prone to be complicated with other risk factors, such as COPD and chronic heart failure. It has been suggested that patients with ≥ 4 risk factors may respond better to NIV (27), which may explain why NIT is more beneficial in older patients. In addition, the duration of prophylactic use of NIT varies by individual, which may affect the efficacy of NIT on EF and reintubation. Further studies are needed to determine the effect of the number of risk factors and duration of intervention on EF.

The respiratory support provided by HFNC is limited, as it may not provide stable positive pressure like NIV (27). However, subgroup analysis of the present study demonstrated that HFNC was noninferior to NIV in reducing EF, which was in accordance with the results of Hernández et al. (12). Numerous studies have confirmed that HFNC is significantly more comfortable and tolerable than NIV (12, 28, 29). In fact, the heating and humidification functions of HFNC allow gas delivery at an optimal humidity, effectively promoting secretion clearance while avoiding side effects such as mucosal dryness (30, 31). Interestingly, we found that HFNC tended to reduce the incidence of delirium, which was in agreement with the findings of Hernández et al. (12) and Stéphan et al. (28). Furthermore, HFNC has a CO_2_ flushing effect on the nasopharyngeal space, thereby decreasing anatomical dead space ventilation and CO_2_ rebreathing (32, 33).

In addition to prophylactic NIT, multivariate logistic regression analysis in our study showed that a higher ROX index before SBT was associated with a reduced risk of EF. The ROX index, defined as the ratio of SpO_2_/FiO_2_ to respiratory rate, is often used as a predictor of reintubation after HFNC failure, with moderate specificity (34, 35). An increasing number of articles have reported the role of ROX index in predicting NIV failure, but there is population heterogeneity, with different time periods for ROX index measurement (36, 37). A retrospective study showed that the ROX index at 6 h after ICU admission helped identify patients with ARDS at risk of NIT failure. Zablockis et al. (38) reported the role of ROX index within 24 h of admission in predicting NIV failure in patients with acute hypoxemic respiratory failure (38). To our knowledge, this study indicated for the first time that pre-SBT ROX index may be associated with the development of EF in high-risk patients. More prospective studies are needed in the future to verify the validity of the ROX index in predicting EF at different time points and to find the best threshold.

In the present study, higher fluid balance 24 h before extubation increased the risk of EF in high-risk patients, which was in agreement with previous studies (39, 40). Weaning-induced pulmonary edema is a common reason for EF (41). And cardiac dysfunction can occur during decannulation owing to increased preload and afterload of the right and left ventricles, triggering EF, especially in high-risk patients (42). Therefore, restricted fluid therapy may be one of the key measures for successful extubation.

Delirium is a common medical problem that is often characterized by transient fluctuations in attention, confusion, and disturbance of thought (43). Delirium has been reported to occur in 50 to 80% of mechanically ventilated patients (44). The incidence of delirium was as high as 54.8% in the high-risk patients included in this study. Older age, ventilator use, and benzodiazepine use increased the risk of delirium in ICU patients (45). This was confirmed in our subgroup analysis, which showed a higher incidence of delirium in patients older than 65 years than in those aged ≤ 65 years. In addition, delirium is a risk factor for EF and reintubation, which was consistent with our results. This may be related to the fact that delirium impedes pulmonary rehabilitation and out-of-bed activities. Not only that, but patients who develop delirium are often treated with benzodiazepines, and the cumulative effect of these sedative drugs can impair mental status after tracheal intubation removal, leading to EF and reintubation.

Although early weaning from MV after successful SBT improves prognosis, EF is inevitable and significantly increases the rate of reintubation. Therefore, it is important to choose an appropriate respiratory strategy to prevent EF, especially for high-risk patients. To prevent EF and increase the success rate of extubation, the modalities of COT, HFNC, and NIV are commonly used to support breathing. In clinical practice, NIV or HFNC could be used prophylactically after planned extubation to reduce the risk of EF in high-risk patients. And NIT is more effective in those older than 65 years. Reducing the incidence of EF and reintubation, and shortening the length of hospital stay are not only beneficial to patients and their families, but also avoid the waste of medical resources. In addition, combinational use of HFNC and NIV seems to be a promising method in post-extubated patients because the addition of HFNC to NIV could, at least theoretically, further improve gas exchange and decrease the work of breathing. In the future, larger sample size randomized controlled trials are needed to explore the effect of combination therapy on extubation failure and reintubation in high-risk patients.

There are several limitations in the study. First, this was a cohort study conducted in a single center, limiting the generalizability of the results. Evaluation methods and parameter settings in different hospitals may affect the effectiveness of NIT on EF. In the future, we will conduct a related multicenter randomized controlled study to further explore the effect of NIT in high-risk patients. Second, due to the nature of retrospective study, there may be potential biases such as selection bias, recalling bias, and confounding factors. These biases may affect the validity of the findings. To address selection bias, we established clear inclusion and exclusion criteria and used uniform and accepted diagnostic criteria. However, PSM was performed in the study to reduce the effect of selection bias and possible confounders between the two groups. The possibility of residual confounding may still exist after PSM. To further control for confounders, other statistical methods can be used, such as stratified analyses or multivariate adjustment analyses, which can help to identify and control for additional confounders. Considering the limited sample size, only two subgroup analyses were performed in this study. Third, although the assessment of SBT is standardized, clinical guidelines are updated over time and the attending physicians make the final decision. Fourth, the small sample size, particularly in the subgroup analysis involving the efficacy of NIV and HFNC, may weaken the strength of the evidence. However, HFNC was proven to be noninferior to NIV in reducing reintubation in relevant multicenter RCTs. Finally, respiratory mechanics parameters such as cough peak expiratory flow (CPEF), peak inspiratory pressure and peak expiratory pressure were missed due to retrospective data collection from medical records. These relevant parameters may be associated with EF. And CPEF is considered to be a useful tool for predicting extubation (46). A CPEF of < 60 L/min was associated with a significantly increased risk of EF (47). The advantage of CPEF is that it is simple, inexpensive, portable, easy to repeat, and has the potential to prevent reintubation. More respiratory mechanical parameters are needed to predict extubation success or failure in the future.

## 5 Conclusion

In conclusion, prophylactic use of NIT following planned extubation is effective in reducing the rate of EF in high-risk patients, especially in those over 65 age of years. HFNC is an alternative treatment to NIV in high-risk patients and increases patient comfort and tolerance. Furthermore, fluid balance, RSBI, ROX index and delirium may be good predictors of EF in high-risk patients.

## Data Availability

The original contributions presented in the study are included in the article/supplementary material, further inquiries can be directed to the corresponding author.
